# Redetermination of Ce[B_5_O_8_(OH)(H_2_O)]NO_3_·2H_2_O

**DOI:** 10.1107/S1600536812016169

**Published:** 2012-04-21

**Authors:** Wei Sun, Teng-Teng Zhu, Biao-Chun Zhao, Ya-Xi Huang, Jin-Xiao Mi

**Affiliations:** a Fujian Provincial Key Laboratory of Advanced Materials, Department of Materials Science and Engineering, College of Materials, Xiamen University, Xiamen 361005, Fujian Province, People’s Republic of China

## Abstract

The crystal structure of Ce[B_5_O_8_(OH)(H_2_O)]NO_3_·2H_2_O, cerium(III) aqua­hydroxidoocta­oxidopenta­borate nitrate dihydrate, has been redetermined from single-crystal X-ray diffraction data. In contrast to the previous determination [Li *et al.* (2003 ▶). *Chem. Mater.* **15**, 2253–2260], the present study reveals the location of all H atoms, slightly different fundamental building blocks (FBBs) of the polyborate anions, more reasonable displacement ellipsoids for all non-H atoms, as well as a model without disorder of the nitrate anion. The crystal structure is built from corrugated polyborate layers parallel to (010). These layers, consisting of [B_5_O_8_(OH)(H_2_O)]^2−^ anions as FBBs, stack along [010] and are linked by Ce^3+^ ions, which exhibit a distorted CeO_10_ coordination sphere. The layers are additionally stabilized *via* O—H⋯O hydrogen bonds between water mol­ecules and nitrate anions, located at the inter­layer space. The [BO_3_(H_2_O)]-group shows a [3 + 1] coordination and is considerably distorted from a tetra­hedral configuration. Bond-valence-sum calculation shows that the valence sum of boron is only 2.63 valence units (v.u.) when the contribution of the water mol­ecule (0.49 v.u.) is neglected.

## Related literature

For a previous structural study of the title compound, see: Li *et al.* (2003 ▶). For the La analogue, see: Li *et al.* (2005 ▶). For the bond-valence method, see: Brown (2002 ▶). For related structures, see: Sun *et al.* (2010 ▶, 2012 ▶). For a review on geometrical parameters of borate groups, see: Zobetz (1982 ▶, 1990 ▶). FBBs in borate crystal chemistry were reviewed by Burns *et al.* (1995 ▶).

## Experimental

### 

#### Crystal data

Ce[B_5_O_8_(OH)(H_2_O)]NO_3_·2H_2_O
*M* *_r_* = 455.24
Monoclinic, 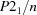

*a* = 6.4438 (12) Å
*b* = 15.572 (3) Å
*c* = 10.745 (2) Å
β = 90.395 (3)°
*V* = 1078.2 (3) Å^3^
*Z* = 4
Mo *K*α radiation
μ = 4.32 mm^−1^
*T* = 173 K
0.30 × 0.12 × 0.04 mm

#### Data collection

Bruker SMART CCD diffractometer
Absorption correction: multi-scan (*SADABS*; Bruker, 2001 ▶) *T*_min_ = 0.357, *T*_max_ = 0.846
6327 measured reflections
2484 independent reflections
2390 reflections with *I* > 2σ(*I*)
*R*_int_ = 0.021

#### Refinement

*R*[*F* ^2^ > 2σ(*F* ^2^)] = 0.026
*wR*(*F* ^2^) = 0.061
*S* = 1.12
2484 reflections
221 parameters
4 restraints
All H-atom parameters refined
Δρ_max_ = 1.40 e Å^−3^
Δρ_min_ = −0.50 e Å^−3^

### 

Data collection: *SMART* (Bruker, 2001 ▶); cell refinement: *SAINT* (Bruker, 2001 ▶); data reduction: *SAINT*; program(s) used to solve structure: *SHELXS97* (Sheldrick, 2008 ▶); program(s) used to refine structure: *SHELXL97* (Sheldrick, 2008 ▶); molecular graphics: *DIAMOND* (Brandenburg, 2011 ▶) and *ATOMS* (Dowty, 2004 ▶); software used to prepare material for publication: *SHELXL97*.

## Supplementary Material

Crystal structure: contains datablock(s) I, global. DOI: 10.1107/S1600536812016169/wm2613sup1.cif

Structure factors: contains datablock(s) I. DOI: 10.1107/S1600536812016169/wm2613Isup2.hkl

Additional supplementary materials:  crystallographic information; 3D view; checkCIF report

## Figures and Tables

**Table:** 

B5—O9	1.406 (5)
B5—O5^i^	1.412 (5)
B5—O8	1.444 (4)
B5—O10	1.637 (5)

**Table:** 

O9—B5—O5^i^	109.8 (3)
O9—B5—O8	114.3 (3)
O5^i^—B5—O8	116.2 (3)
O9—B5—O10	107.1 (3)
O5^i^—B5—O10	107.3 (3)
O8—B5—O10	101.1 (3)

Symmetry code: (i) 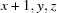.

**Table 2:** Hydrogen-bond geometry (Å, °)

*D*—H⋯*A*	*D*—H	H⋯*A*	*D*⋯*A*	*D*—H⋯*A*
O1—H1⋯O3^ii^	0.88 (6)	1.75 (6)	2.622 (3)	173 (6)
O10—H2⋯O13^i^	0.85 (2)	1.89 (3)	2.705 (4)	160 (6)
O10—H3⋯O12	0.85 (2)	1.85 (2)	2.700 (4)	173 (6)
O15—H4⋯O4	0.84 (7)	2.21 (7)	2.967 (4)	150 (6)
O15—H4⋯O10^iii^	0.84 (7)	2.50 (6)	3.041 (4)	123 (6)
O15—H5⋯O13^iv^	0.84 (7)	2.10 (7)	2.912 (4)	164 (6)
O15—H5⋯O12^iv^	0.84 (7)	2.48 (7)	3.093 (4)	131 (5)
O14—H6⋯O2^iv^	0.81 (2)	2.01 (3)	2.756 (4)	153 (6)

Symmetry codes: (i) 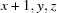; (ii) 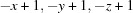; (iii) 
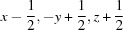
; (iv) 
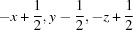
.

**Fig. 1.**
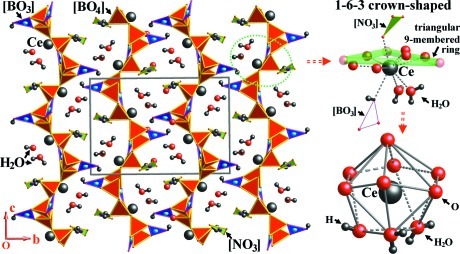
The crystal structure of Ce[B5O8(OH)(H2O)][NO3].2H2O projected onto (100). [BO4] groups are drawn as red tetrahedra; [BO3] as blue triangular groups; [NO3] as green triangular groups; Ce as black spheres; O as red spheres; H as small black spheres). The inset on the right represents the coordination environment of the Ce atom in a 1–6–3 crown-shaped polyhedron.

**Fig. 2.**
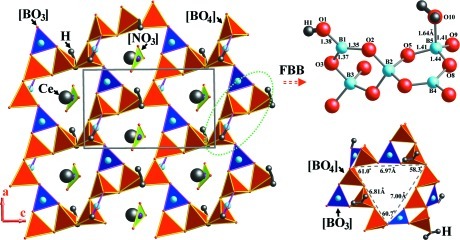
The Crystal structure of Ce[B5O8(OH)(H2O)][NO3].2H2O projected onto (010). Top right: the fundamental building block (FBB) of [B5O8(OH)(H2O)]; Bottom right: the 9-membered ring with a nearly equilateral triangular motif.

**Fig. 3.**
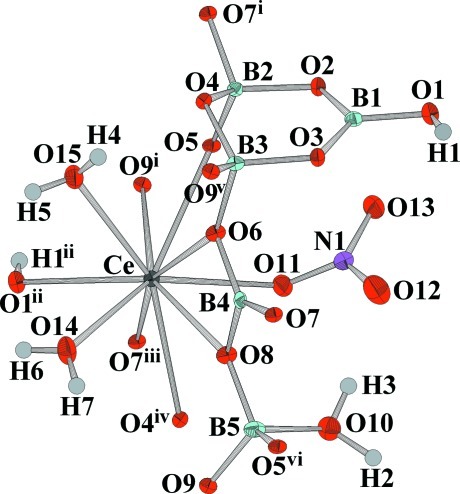
Coordination environment of the cerium atom with displacement ellipsoids drawn at the 50% probability level. [Symmetry codes: (i) x–1, y, z; (ii) –x + 1/2, y–1/2, –z + 1/2; (iii) x–1/2, –y + 1/2, z–1/2; (iv) x + 1/2, –y + 1/2, z–1/2; (v) x–1/2, –y + 1/2, z + 1/2; (vi) x + 1, y, z.]

## supplementary crystallographic information

### Comment

Borate compounds have been extensively studied due to their diverse structural chemistry and successful industry applications. The title compound, Ce[B_5_O_8_(OH)(H_2_O)][NO_3_]^.^2H_2_O, has already been prepared and reported by Li *et al.* (2003) with a structure model that describes disordered oxygen positions of the nitrate anion, and where the hydrogen positions could not be determined even in the ordered model of the La analogue (Li *et al.*, 2005). Herein we report the redetermined crystal structure based on single-crystal X-ray diffraction data of Ce[B_5_O_8_(OH)(H_2_O)][NO_3_]^.^2H_2_O with the location of all H atoms, slightly different fundamental building blocks (FBBs) of the polyborate anions, more reasonable displacement ellipsoids for all non-hydrogen atoms as well as a model without disorder of the nitrate anion.

The crystal structure of Ce[B_5_O_8_(OH)(H_2_O)][NO_3_]^.^2H_2_O is built from corrugated polyborate layers parallel to (010) which stack along the [010] direction (Figs 1, 2). The polyborate layer is made up of zigzag borate branched chains running along the [100] direction, and consists of B_5_O_8_(OH)(H_2_O) as fundamental building blocks (FBBs) (Burns *et al.*, 1995). Each FBB consists of two [BO_4_] tetrahedra, one [BO_3_(H_2_O)] tetrahedron, one [BO_3_] triangle, and a planar trigonal [BO_2_(OH)] group. The borate groups of the FBB, except for the [BO_2_(OH)] group, form the backbone of the single infinite chain, one side of which is decorated by flanking trigonal planar [B1O_2_(OH)] groups. These zigzag infinite chains are, *via* symmetry operations (*i.e.* with each rotated by 180° with respect to the adjacent chains), alternately arranged with the flanking [BO_2_(OH)] groups up and down, respectively, and fused *via* common O-vertices, resulting in a two-dimensional corrugated layer with 9-membered rings within the layer. The 9-membered ring has a nearly equilateral triangular motif with edge lengths of about 7.0 Å (Fig. 2) as that in [Ce(B_4_O_6_(OH)_2_)Cl] (Sun *et al.*, 2012). The Ce^3+^ ion resides at the center of the 9-membered rings and adopt a distorted 10-coordination to the surrounding oxygen atoms to form a 1–6–3 crown-shaped polyhedron (Figs. 1, 3), six of them coming from the nearly planar 9-membered ring in the middle, one from a triangular [NO_3_] anion on the top, one from an OH group (originating from a triangular [BO_2_(OH)] group from the next layer) and two from water molecules at the botton. The water molecules and the nitrate [NO_3_] groups, located at the interlayer space, additionally stabilize the structural set-up of the title nitrate borate compound, *via* their O–H···O hydrogen bonds (Table 2). In the present model, H-atom H7 (attached to O14) has no acceptor atom, and none of alternative approximate positions found in difference Fourier maps for H7 were reliable because they were too close to the Ce^3+^ ion. All hydrogen atoms except for H7 point to the backbone of the polyborate layers, whereas H-atom H7 points to the [100] direction (*i.e.* O14–H7 parallel to the polyborate layers).

In contrast to the previous report (Li *et al.*, 2003), one of the 3-coordinated boron atoms with B–O distances less than 1.38 Å (denoted as B5 in this paper) was altered to be '3 + 1' coordinated to three surrounding O-atoms and a water molecule forming a highly distorted tetrahedral [BO_3_(H_2_O)]-group in the present description. The highly distorted tetrahedral [BO_3_(H_2_O)] group has quite a long B—O bond distance (*d*(B–OH_2_) = 1.637 (5) Å) and three normal distances (*d*(B–O) = 1.406 (5) to 1.444 (5) Å; Table 1), as observed in its La counterpart (Li *et al.*, 2005) and previous reviews on the crystal chemistry of borates (Zobetz, 1982, 1990). This may be attributed to the fact that the water molecule strongly attracts the boron atom to offset from the trigonal plane. Additionally, bond valence sum calculations (Brown, 2002) also support this assumption because the valence sum of boron is only 2.63 *v.u.* if not considering the contribution of water molecule (0.49 *v.u.*).

### Experimental

During our systematically investigation on rare earth borates (Sun *et al.*, 2010; Sun *et al.*, 2012), the title compound, Ce[B_5_O_8_(OH)(H_2_O)][NO_3_]^.^2H_2_O, was synthesized by using molten boric acid as flux which has been firstly described by Li *et al.* (2003). Typically, a mixture of Ce(NO_3_)_3_^.^6H_2_O (1.00 g) and H_3_BO_3_ (3.00 g) with molar ratio of Ce:B = 1:21 was prepared by thoroughly homogeneous grinding and transferred into a Teflon-lined stainless-steel autoclave (30 ml in volume), then heated to and kept at 513 K for three days. Transparent, colorless crystals of the title compound were obtained by filtration, rinsed with deionized water for several times, and dried in a desiccators. The phases of the as-prepared solid products were identified by powder X-ray diffraction (PXRD) analyses. Optical microscopy was used to check the selected crystals proper for single-crystal X-ray diffraction while their chemical compositions were examined by use of an Energy Dispersive X-ray Spectrometer (EDX) (Oxford Instruments). Scanning electron microscopy (SEM) was used to document the crystal morphologies.

### Refinement

Initially, all hydrogen positions were located from difference Fourier maps and refined freely. Then a common variable was used for the isotropic atomic displacement parameters (*U*_iso_) of all hydrogen atoms while their atomic coordinates were refined. After refinement the O–H bond lengths of 2 water molecules (*i.e.* H3–O10–H2 and H7–O14–H6) became improper, soft restraints on *U*_iso_ and on bond lengths (*d*(O–H) = 0.82 (2) Å) were applied.

### Crystal data

**Table:**

Ce[B_5_O_8_(OH)(H_2_O)]NO_3_·2H_2_O	*F*(000) = 868
*M**_r_* = 455.24	*D*_x_ = 2.805 Mg m^−^^3^
Monoclinic, *P*2_1_/*n*	Mo *K*α radiation, λ = 0.71073 Å
Hall symbol: -P 2yn	Cell parameters from 6327 reflections
*a* = 6.4438 (12) Å	θ = 2.3–28.2°
*b* = 15.572 (3) Å	µ = 4.32 mm^−^^1^
*c* = 10.745 (2) Å	*T* = 173 K
β = 90.395 (3)°	Prism, colorless
*V* = 1078.2 (3) Å^3^	0.30 × 0.12 × 0.04 mm
*Z* = 4	

### Data collection

**Table:**

Bruker SMART CCD diffractometer	2484 independent reflections
Radiation source: fine-focus sealed tube	2390 reflections with *I* > 2σ(*I*)
Graphite monochromator	*R*_int_ = 0.021
1800 images,φ=0, 90, 180 degree, and Δω=0.3 degree, χ= 54.74 degree scans	θ_max_ = 28.2°, θ_min_ = 2.3°
Absorption correction: multi-scan (*SADABS*; Bruker, 2001)	*h* = −8→6
*T*_min_ = 0.357, *T*_max_ = 0.846	*k* = −20→20
6327 measured reflections	*l* = −13→14

### Refinement

**Table:**

Refinement on *F*^2^	Primary atom site location: structure-invariant direct methods
Least-squares matrix: full	Secondary atom site location: difference Fourier map
*R*[*F*^2^ > 2σ(*F*^2^)] = 0.026	Hydrogen site location: difference Fourier map
*wR*(*F*^2^) = 0.061	All H-atom parameters refined
*S* = 1.12	*w* = 1/[σ^2^(*F*_o_^2^) + (0.0269*P*)^2^ + 2.2495*P*] where *P* = (*F*_o_^2^ + 2*F*_c_^2^)/3
2484 reflections	(Δ/σ)_max_ < 0.001
221 parameters	Δρ_max_ = 1.40 e Å^−^^3^
4 restraints	Δρ_min_ = −0.50 e Å^−^^3^

### Special details

**Table:**

Geometry. All e.s.d.'s (except the e.s.d. in the dihedral angle between two l.s. planes) are estimated using the full covariance matrix. The cell e.s.d.'s are taken into account individually in the estimation of e.s.d.'s in distances, angles and torsion angles; correlations between e.s.d.'s in cell parameters are only used when they are defined by crystal symmetry. An approximate (isotropic) treatment of cell e.s.d.'s is used for estimating e.s.d.'s involving l.s. planes.
Refinement. Refinement of *F*^2^ against ALL reflections. The weighted *R*-factor *wR* and goodness of fit *S* are based on *F*^2^, conventional *R*-factors *R* are based on *F*, with *F* set to zero for negative *F*^2^. The threshold expression of *F*^2^ > σ(*F*^2^) is used only for calculating *R*-factors(gt) *etc*. and is not relevant to the choice of reflections for refinement. *R*-factors based on *F*^2^ are statistically about twice as large as those based on *F*, and *R*- factors based on ALL data will be even larger.

### Fractional atomic coordinates and isotropic or equivalent isotropic displacement parameters (Å^2^)

**Table:**

	*x*	*y*	*z*	*U*_iso_*/*U*_eq_	
Ce1	0.35178 (3)	0.201751 (12)	0.139251 (16)	0.00786 (7)	
B1	0.2409 (6)	0.4622 (3)	0.4100 (3)	0.0094 (7)	
B2	0.0226 (6)	0.3373 (2)	0.3613 (3)	0.0083 (7)	
B3	0.3600 (6)	0.3163 (2)	0.4625 (3)	0.0081 (7)	
B4	0.6568 (6)	0.2918 (2)	0.3206 (4)	0.0088 (7)	
B5	0.9081 (6)	0.2807 (3)	0.1513 (4)	0.0125 (7)	
N1	0.3614 (5)	0.4247 (2)	0.0851 (3)	0.0166 (6)	
O1	0.2738 (4)	0.54996 (15)	0.4121 (2)	0.0116 (5)	
O2	0.0638 (4)	0.43136 (15)	0.3587 (2)	0.0095 (5)	
O3	0.3906 (3)	0.41051 (15)	0.4608 (2)	0.0096 (5)	
O4	0.1407 (4)	0.29595 (14)	0.4617 (2)	0.0074 (5)	
O5	0.0654 (4)	0.29975 (15)	0.2391 (2)	0.0088 (5)	
O6	0.4540 (4)	0.27987 (15)	0.3470 (2)	0.0094 (5)	
O7	0.8026 (3)	0.32384 (15)	0.3991 (2)	0.0086 (4)	
O8	0.7032 (4)	0.26620 (16)	0.2007 (2)	0.0106 (5)	
O9	0.9770 (4)	0.21643 (15)	0.0695 (2)	0.0088 (5)	
O10	0.8694 (4)	0.36739 (18)	0.0682 (3)	0.0182 (6)	
O11	0.3550 (4)	0.35075 (17)	0.0378 (2)	0.0180 (5)	
O12	0.5235 (4)	0.4534 (2)	0.1344 (3)	0.0285 (7)	
O13	0.2040 (4)	0.47208 (17)	0.0797 (3)	0.0226 (6)	
O14	0.6030 (4)	0.08603 (18)	0.2165 (3)	0.0195 (6)	
O15	0.1547 (5)	0.13445 (19)	0.3154 (3)	0.0190 (6)	
H1	0.388 (9)	0.559 (4)	0.455 (5)	0.056 (7)*	
H2	0.967 (7)	0.402 (3)	0.054 (5)	0.056 (7)*	
H3	0.767 (6)	0.398 (3)	0.090 (5)	0.056 (7)*	
H4	0.160 (9)	0.167 (5)	0.378 (6)	0.056 (7)*	
H5	0.189 (9)	0.084 (4)	0.331 (6)	0.056 (7)*	
H6	0.582 (9)	0.0349 (15)	0.211 (6)	0.056 (7)*	
H7	0.729 (3)	0.088 (4)	0.223 (6)	0.056 (7)*	

### Atomic displacement parameters (Å^2^)

**Table:**

	*U*^11^	*U*^22^	*U*^33^	*U*^12^	*U*^13^	*U*^23^
Ce1	0.00734 (11)	0.00863 (11)	0.00758 (11)	−0.00024 (6)	−0.00198 (7)	−0.00037 (6)
B1	0.0099 (17)	0.0114 (18)	0.0070 (16)	−0.0001 (14)	0.0018 (13)	−0.0007 (13)
B2	0.0058 (16)	0.0100 (18)	0.0090 (16)	−0.0002 (13)	−0.0010 (13)	−0.0007 (13)
B3	0.0069 (17)	0.0116 (18)	0.0057 (16)	0.0008 (13)	−0.0009 (13)	0.0009 (13)
B4	0.0102 (18)	0.0058 (17)	0.0105 (17)	0.0010 (13)	−0.0005 (13)	0.0031 (13)
B5	0.0089 (18)	0.0160 (19)	0.0125 (18)	−0.0001 (15)	0.0008 (14)	−0.0024 (15)
N1	0.0140 (15)	0.0147 (16)	0.0213 (16)	0.0002 (12)	0.0054 (12)	0.0013 (12)
O1	0.0118 (12)	0.0092 (12)	0.0138 (12)	0.0005 (9)	−0.0059 (9)	−0.0001 (9)
O2	0.0092 (11)	0.0083 (12)	0.0111 (11)	−0.0004 (9)	−0.0030 (9)	0.0009 (9)
O3	0.0079 (11)	0.0079 (12)	0.0128 (11)	−0.0003 (9)	−0.0029 (9)	0.0005 (9)
O4	0.0068 (11)	0.0094 (12)	0.0061 (10)	−0.0006 (8)	−0.0021 (8)	0.0008 (8)
O5	0.0054 (11)	0.0121 (12)	0.0089 (11)	0.0002 (8)	−0.0005 (9)	−0.0012 (8)
O6	0.0079 (11)	0.0143 (12)	0.0058 (10)	−0.0018 (9)	−0.0024 (9)	−0.0018 (9)
O7	0.0065 (11)	0.0118 (12)	0.0074 (10)	−0.0010 (9)	−0.0011 (8)	−0.0011 (9)
O8	0.0088 (11)	0.0156 (13)	0.0074 (11)	−0.0018 (9)	−0.0022 (9)	−0.0028 (9)
O9	0.0075 (11)	0.0108 (12)	0.0081 (11)	0.0011 (9)	−0.0020 (9)	−0.0005 (9)
O10	0.0170 (14)	0.0185 (14)	0.0192 (13)	0.0014 (10)	−0.0022 (11)	0.0025 (11)
O11	0.0237 (14)	0.0128 (13)	0.0176 (12)	0.0006 (10)	0.0057 (11)	−0.0008 (10)
O12	0.0180 (15)	0.0232 (16)	0.0441 (19)	0.0012 (12)	−0.0086 (13)	−0.0089 (13)
O13	0.0182 (14)	0.0142 (14)	0.0354 (16)	0.0035 (11)	0.0036 (12)	0.0019 (12)
O14	0.0150 (13)	0.0127 (13)	0.0307 (15)	0.0004 (10)	−0.0074 (11)	−0.0003 (11)
O15	0.0287 (16)	0.0122 (13)	0.0160 (13)	−0.0008 (11)	0.0068 (11)	−0.0002 (11)

### Geometric parameters (Å, º)

**Table:**

Ce1—O15	2.515 (3)	B3—O3	1.481 (4)
Ce1—O9^i^	2.534 (2)	B3—O6	1.496 (4)
Ce1—O14	2.557 (3)	B4—O6	1.352 (4)
Ce1—O1^ii^	2.558 (2)	B4—O7	1.353 (4)
Ce1—O8	2.559 (2)	B4—O8	1.383 (4)
Ce1—O11	2.564 (3)	B5—O9	1.406 (5)
Ce1—O6	2.622 (2)	B5—O5^vi^	1.412 (5)
Ce1—O7^iii^	2.628 (2)	B5—O8	1.444 (4)
Ce1—O5	2.629 (2)	B5—O10	1.637 (5)
Ce1—O4^iv^	2.675 (2)	N1—O12	1.250 (4)
B1—O2	1.352 (4)	N1—O13	1.255 (4)
B1—O3	1.367 (4)	N1—O11	1.260 (4)
B1—O1	1.383 (5)	O1—H1	0.88 (6)
B2—O4	1.465 (4)	O10—H2	0.85 (2)
B2—O5	1.466 (4)	O10—H3	0.85 (2)
B2—O2	1.489 (4)	O15—H4	0.84 (7)
B2—O7^i^	1.492 (4)	O15—H5	0.84 (7)
B3—O4	1.448 (4)	O14—H6	0.81 (2)
B3—O9^v^	1.462 (4)	O14—H7	0.81 (2)
			
O15—Ce1—O9^i^	77.00 (9)	O2—B1—O1	119.1 (3)
O15—Ce1—O14	77.52 (10)	O3—B1—O1	117.9 (3)
O9^i^—Ce1—O14	139.43 (8)	O4—B2—O5	112.6 (3)
O15—Ce1—O1^ii^	67.46 (9)	O4—B2—O2	110.8 (3)
O9^i^—Ce1—O1^ii^	73.74 (8)	O5—B2—O2	109.9 (3)
O14—Ce1—O1^ii^	67.52 (8)	O4—B2—O7^i^	103.2 (3)
O15—Ce1—O8	114.82 (9)	O5—B2—O7^i^	111.9 (3)
O9^i^—Ce1—O8	151.72 (7)	O2—B2—O7^i^	108.3 (3)
O14—Ce1—O8	68.62 (8)	O4—B3—O9^v^	115.2 (3)
O1^ii^—Ce1—O8	134.13 (8)	O4—B3—O3	110.3 (3)
O15—Ce1—O11	134.64 (9)	O9^v^—B3—O3	106.7 (3)
O9^i^—Ce1—O11	78.63 (8)	O4—B3—O6	108.2 (3)
O14—Ce1—O11	140.26 (9)	O9^v^—B3—O6	108.1 (3)
O1^ii^—Ce1—O11	138.42 (8)	O3—B3—O6	108.1 (3)
O8—Ce1—O11	75.24 (8)	O6—B4—O7	126.0 (3)
O15—Ce1—O6	71.22 (9)	O6—B4—O8	111.8 (3)
O9^i^—Ce1—O6	116.31 (7)	O7—B4—O8	122.2 (3)
O14—Ce1—O6	84.02 (8)	O9—B5—O5^vi^	109.8 (3)
O1^ii^—Ce1—O6	133.62 (7)	O9—B5—O8	114.3 (3)
O8—Ce1—O6	51.82 (7)	O5^vi^—B5—O8	116.2 (3)
O11—Ce1—O6	86.53 (8)	O9—B5—O10	107.1 (3)
O15—Ce1—O7^iii^	128.13 (9)	O5^vi^—B5—O10	107.3 (3)
O9^i^—Ce1—O7^iii^	67.35 (7)	O8—B5—O10	101.1 (3)
O14—Ce1—O7^iii^	106.49 (8)	O12—N1—O13	118.8 (3)
O1^ii^—Ce1—O7^iii^	67.17 (7)	O12—N1—O11	121.5 (3)
O8—Ce1—O7^iii^	114.42 (7)	O13—N1—O11	119.6 (3)
O11—Ce1—O7^iii^	73.79 (8)	Ce1^vii^—O1—H1	103 (4)
O6—Ce1—O7^iii^	159.08 (8)	B1—O1—H1	107 (4)
O15—Ce1—O5	64.87 (9)	B1—O2—B2	119.4 (3)
O9^i^—Ce1—O5	53.01 (7)	B1—O3—B3	119.6 (3)
O14—Ce1—O5	136.14 (8)	B3—O4—B2	114.2 (3)
O1^ii^—Ce1—O5	113.74 (8)	B5^i^—O5—B2	122.9 (3)
O8—Ce1—O5	106.81 (7)	B4—O6—B3	121.3 (3)
O11—Ce1—O5	69.88 (8)	B4—O7—B2^vi^	122.6 (3)
O6—Ce1—O5	63.65 (7)	B4—O8—B5	120.1 (3)
O7^iii^—Ce1—O5	114.11 (7)	B5—O9—B3^iv^	125.2 (3)
O15—Ce1—O4^iv^	154.12 (8)	B5—O10—H2	121 (4)
O9^i^—Ce1—O4^iv^	117.02 (7)	B5—O10—H3	115 (4)
O14—Ce1—O4^iv^	78.40 (8)	H2—O10—H3	106 (6)
O1^ii^—Ce1—O4^iv^	94.54 (7)	N1—O11—Ce1	131.0 (2)
O8—Ce1—O4^iv^	63.89 (7)	Ce1—O14—H6	124 (4)
O11—Ce1—O4^iv^	71.15 (7)	Ce1—O14—H7	129 (5)
O6—Ce1—O4^iv^	115.40 (7)	H6—O14—H7	102 (6)
O7^iii^—Ce1—O4^iv^	51.80 (7)	Ce1—O15—H4	110 (4)
O5—Ce1—O4^iv^	141.00 (7)	Ce1—O15—H5	114 (4)
O2—B1—O3	123.0 (3)	H4—O15—H5	113 (6)

Symmetry codes: (i) *x*−1, *y*, *z*; (ii) −*x*+1/2, *y*−1/2, −*z*+1/2; (iii) *x*−1/2, −*y*+1/2, *z*−1/2; (iv) *x*+1/2, −*y*+1/2, *z*−1/2; (v) *x*−1/2, −*y*+1/2, *z*+1/2; (vi) *x*+1, *y*, *z*; (vii) −*x*+1/2, *y*+1/2, −*z*+1/2.

### Hydrogen-bond geometry (Å, º)

**Table:**

*D*—H···*A*	*D*—H	H···*A*	*D*···*A*	*D*—H···*A*
O1—H1···O3^viii^	0.88 (6)	1.75 (6)	2.622 (3)	173 (6)
O10—H2···O13^vi^	0.85 (2)	1.89 (3)	2.705 (4)	160 (6)
O10—H3···O12	0.85 (2)	1.85 (2)	2.700 (4)	173 (6)
O15—H4···O4	0.84 (7)	2.21 (7)	2.967 (4)	150 (6)
O15—H4···O10^v^	0.84 (7)	2.50 (6)	3.041 (4)	123 (6)
O15—H5···O13^ii^	0.84 (7)	2.10 (7)	2.912 (4)	164 (6)
O15—H5···O12^ii^	0.84 (7)	2.48 (7)	3.093 (4)	131 (5)
O14—H6···O2^ii^	0.81 (2)	2.01 (3)	2.756 (4)	153 (6)

Symmetry codes: (ii) −*x*+1/2, *y*−1/2, −*z*+1/2; (v) *x*−1/2, −*y*+1/2, *z*+1/2; (vi) *x*+1, *y*, *z*; (viii) −*x*+1, −*y*+1, −*z*+1.
